# The bio-politics of subsistence: nutritional lock-in, human capital, and civic capacity

**DOI:** 10.3389/fpubh.2026.1937382

**Published:** 2026-10-15

**Authors:** Muhammad Shahid, Jiayi Song, Abid Rehman, Hafiz Muhammad Naveed, Zaiba Ali, Serhat Yuksel, Hasan Dinçer

**Affiliations:** 1 College of Management, Shenzhen University, Shenzhen, Guangdong, China; 2 Department of Family Medicine, Faculty of Medicine and Health Sciences, McGill University, Montreal, QC, Canada; 3 College of Economics and Management, South China Agricultural University, Guangzhou, China; 4 School of Management Science and Engineering, Shandong University of Finance and Economics, Shandong, Jinan, China; 5 Department of Management, College of Business Administration, Princess Nourah Bint Abdulrahman University, Riyadh, Saudi Arabia; 6 School of Business, Istanbul Medipol University, Istanbul, Türkiye; 7 Clinic of Economics, Azerbaijan State University of Economics (UNEC), Baku, Azerbaijan; 8 Department of Economics and Management, Khazar University, Baku, Azerbaijan; 9 University College, Korea University, Seoul, Republic of Korea

**Keywords:** civic capacity, food policy, human capital, nutrition, nutritional lock-in, Pakistan, political economy, poverty traps

## Abstract

**Background:**

Why do politically salient food policies persist even when they may generate long-term developmental costs? This paper develops and empirically examines the concept of nutritional lock-in, a framework in which food-policy reforms can alter early-life nutritional conditions and, in turn, shape subsequent human-capital outcomes and dimensions of civic capacity.

**Method:**

We study Pakistan's 2008–2010 wheat-subsidy reforms, exploiting district-level variation in pre-reform exposure to the public distribution system in a difference-in-differences framework. Additionally, study also used three waves of the Pakistan Demographic and Health Survey (2006–07, 2012–13, and 2017–18) to find the impact on children heights-for-age.

**Results:**

Study find that children in districts with greater pre-reform exposure experienced a decline of approximately 0.28 standard deviations in height-for-age following the reforms. The estimated effect remains similar after controlling for child, maternal, and household characteristics and in sibling fixed-effects specifications. Complementary analyses indicate substantial changes in dietary composition, including lower dietary diversity and reduced consumption of several nutrient-rich food groups. District-cohort analyses further show that early-life nutritional shocks are negatively associated with later standardized mathematics achievement, although these estimates are correlational. Similarly, women in more highly exposed districts exhibit lower scores on measures of autonomy, mobility, media exposure, and community participation, which we interpret as proxies for civic capacity rather than direct measures of political behavior.

**Conclusion:**

The study findings are consistent with the nutritional lock-in framework: food-policy reforms can have consequences extending beyond immediate fiscal or consumption effects to child nutrition, human-capital formation, and dimensions of civic capacity. However, the data do not identify the complete causal pathway from childhood nutrition to political accountability or policy persistence. The findings therefore motivate a broader view of food policy as a component of long-run human-capital and institutional development.

## 1 Introduction

The persistence of destructive policies despite years of evidence that they inhibit development has long puzzled political economists (1, 2). One area where the contradiction is clearest is in agricultural sectors across developing countries that squander large financial resources on mass food subsidy programs that not only do little to meet, and are often detrimental to achieving good outcomes for people living with malnourished (3, 4). The situation in Pakistan is a prime example of this paradox: whereas Pakistan runs one of the largest wheat procurement and distribution systems in the world and spends 1.2% of its Gross Domestic Product (GDP) on subsidies for wheat sale (5), it continues to face some of the highest child stunting rates globally (6–13). Recent evidence suggests that 38.2% of under-five children in Pakistan suffer from stunted growth and development, as reported by the 2018 Pakistan Demographic and Health Survey (14). Further, previous research has already shown that poor socio-economic and human development is generally highest in families with undernourished children. This predicament usually leads to this family being trapped in an inter-generational poverty (15–17).

This paper provides and tests empirically the theory of “nutritional lock-in”— hypothesized process in which elite-driven food policies create biological constraints on child development that subsequently undermine the human capital necessary for political accountability, thereby perpetuating the very policies that caused the initial harm—to explain a puzzling coexistence of high food subsidies with widespread malnutrition. The conceptual framework describes a vicious circle in which the elite-driven food policy decisions create biological constraints on child development. These in turn undermine the basic human capital required for political accountability (18), and provide reinforcement to the very policies that cause the impairment (19, 20). This synthesis connects the political economy literature with the developmental origins of health and disease (21–23) by showing how policies turn into a set of epigenetic/biological inputs across generations through their impacts on early life nutrition (24, 25).

This paper contributes theoretically by extending models of institutional persistence with a contemporary biological mechanism that may sustain inefficient equilibria (19). While historical institutionalism emphasizes the importance of path dependence driven by colonial legacies (26), this framework shows that current policies create biological constraints that entrenched power asymmetries. This approach extends Sen's (27) entitlement framework to argue that political structures do not simply govern current food security, but also determine the capacity for future wellbeing through biological pathways (27). This link contributes to deeper theorizing on capability formation (28–30).

Empirically, the paper uses the 2008–2010 wheat subsidy reforms in Pakistan as a natural experiment. These reforms, adopted under the compulsion of IMF following global price shocks, provide potentially exogenous variation in subsidy levels (31). Utilizing a difference-in-differences strategy, the analysis contrasts nutritional, educational, and attitudinal measures between districts with differing pre-reform exposure to the public distribution system. Our results show large negative effects of reduced subsidies on child height-for-age, which are associated with lower human capital attainment and weak civic participation among affected cohorts (32, 33). The empirical approach introduces a novelty in using differences in physical infrastructure, which means we can avoid measuring directly the effect of elite influence as well as response to common adjustment problems when analyzing political economy of food systems (34).

Our research builds on existing findings and advances the field in two primary respects. First, we constructed an analytical framework of “nutritional lock-in,” which links three factors together: food-related policies enacted by various countries, the nutritional status of individuals during early childhood, and the formation process of individual human capital. The core purpose of establishing this framework is to highlight the long-term developmental impacts that food subsidies can impose on people (35, 36). Second, we obtained quasi-experimental evidence by leveraging the wheat-subsidy reforms implemented in Pakistan from 2008 to 2010; this evidence specifies the effects of changes in people's access to food on the nutritional status of children. After completing this part of the research, we further explored the associations between nutritional disparities among these children and their academic performance and civic competence in adulthood (37). Unfortunately, limited by the datasets available to us, these analyses of subsequent impacts only propose correlational possibilities, and cannot prove the existence of a direct causal relationship between nutritional disparities and their later academic outcomes and abilities (38). Overall, while all the evidence we have found can support some elements within this “nutritional lock-in” framework, it cannot fully reproduce the complete cycle that starts from changes in nutritional status and extends to the long-term sustainability of related policies.

## 2 Conceptual framework: nutritional lock-in

The rationale relies on three literatures, which are well-established: political economy of redistribution and investment in human capital literature; and developmental origins of health and disease. Out of context, the main message which emerges from these views is that food policies measured against the constricted yardsticks of fiscal discipline or short-term economic efficiency do less well than they are conventionally thought to. However, these policies remain in place because of their political function and biological effects which erode the conditions for political safeguards against elite action over time (19, 39).

Before doing so, however, we need to make a distinction between two closely related but different terms that you will find repeatedly in this paper. When we refer to “political accountability” it is the capacity of citizens through institutional channels (voting, aggregate petitioning and civic oversight mechanisms) to keep track of elite behavior so they can punish or reward through their choices. When we think of “civic capacity” or “civic engagement,” we mean a wider set of social, cognitive, and informational resources which all facilitate the political accountability that is needed for responsible governance: media exposure; mobility; community participation. Our empirical analysis provides direct proxies for civic capacity (empowerment indices of women—autonomy, mobility, and media access). We acknowledge a limitation with respect to the absence of direct measures (e.g., voter turnout) in our main data on measures of political accountability.

From the political economy perspective, it builds on models of redistributive politics in which elites create policies that seek to remain in control while simultaneously extracting economic rents. In agrarian contexts, the subsidies to staple foods have also been 2-fold—it is a source of income support for politically influential farmers while simultaneously providing cheap calories for the urban masses that are someone at the top will equate in political value (35, 40). This tension forms a “coalition of the disparate,” wherein control group members are only connected through how much subsidy they need, making reform difficult (20, 41). This framework is also informed by insights from institutional economics, which shows how the structures of policy create locked-in vested interests and a path-dependence pathway (42).

The human capital dimension includes major insights about early childhood investments from the economics of skill formation. This paradigm emphasizes the very high returns to investments in nutrition during critical periods of development (25, 43). It also illustrates the extent to which incentives for unhelpful investments arising from policy distortion of relative food prices may be, or are being avoided (44, 45). This conversation sits within a broader literature that examines mechanisms by which human capital investment can experience market failures resulting in poverty traps (46, 47). The model is based on Becker's (48) canonical human capital model with nutrition as a vital input to building capability (48).

The biological pathway is founded on the belief that early life exposures have long-term implications for health, cognitive performance and ultimately economic productivity (33, 49). Epigenetics offers a mechanistic framework where early nutritional climate elicits long-lasting responses in gene expression and function (50). This latest take expands on those views through a public health lens by detailing how political determinants, and more specifically food policies biologically impact humans in future generations from the time of conception onward. This conception is consistent with evidence stemming from biological embedding of social experiences which suggest a deep mechanism through which socio-political factors may ultimately affect biological outcomes (51, 52).

The distinctive contribution of this study is conceptualization of feedbacks from human capital to political accountability. Extending existing models of political agency (53, 54), we contend that citizens' capacity to hold elites accountable is deeply influenced by cognitive abilities, information processing skills and collective action capacities—factors which are markedly shaped by early nutritional investments (33, 55). In this formulation, even when subsidies ensure basic caloric availability, the selective nature of distribution and the nutritional deficits among vulnerable populations ultimately weaken the human capital required for effective political oversight. This result is directly related to research on the effect of human capital for political participation and democracy quality (56, 57).

The equilibrium is described following models of institutional persistence with complementarities (58, 59). Our model depicts how multiple steady states of development can coexist: a low-level trap with high subsidies, malnourishment, low human capital and weak accountability and a high-level equilibrium with moderate subsidies, good nutrition, high human capital and strong accountability. Users may experience one or the other of these possible outcomes, depending on historical context—including colonial historical elements [i.e., land tenure and agricultural policy] (26), and transitions between them require changes to intersecting sets of conditions. This view accords with literature on multiple equilibria in development (60, 61).

The framework generates a number of testable implications that guide the empirical evaluation. First, adverse shocks to subsidies on staples are anticipated to result in worsening child nutrition outcomes, some of which will occur disproportionately in those regions where the subsidized system is heavily used. Second, such negative consequences should be most pronounced in early childhood, which is a crucial period for nutritional investment and when its elasticities appear higher. Third, early childhood nutritional shocks may have long-term effects on educational attainment. Fourth, regions suffering heavier nutritional shocks should show declines in civic capacity in later periods. Fifth, the effects are likely to fall most heavily on poor households, who have little room in their budget for substituting other foods. The nutrition lock-in mechanism (Figure 1) may generalize beyond Pakistan to those settings in which food accessibility depends on political factors, and where early-life nutritional status is sensitive to price volatility.

**Figure 1:**
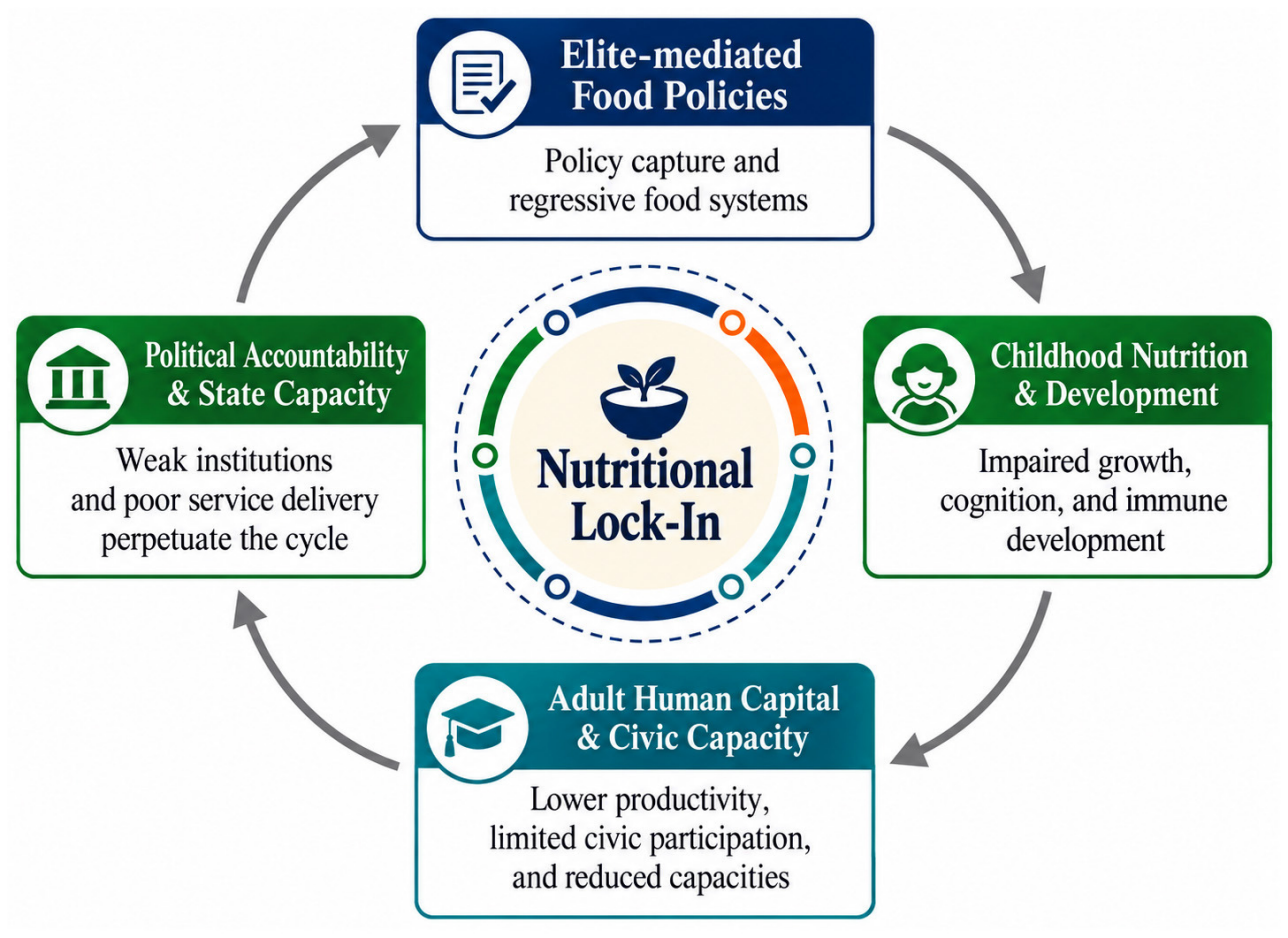
The Self-reinforcing equilibrium of nutritional lock-in.

## 3 Institutional context and empirical strategy

Pakistan's wheat industry exists in a “canalized” system, as defined by agricultural economists which refers to the framework in which there is critical government involvement at multiple stages of production and distribution (62). The agriculture cycle begins when provincial governments announce MSPs (minimum support prices), which are meant to be a safety net for farmers. After that, Government agencies like PACCO (Pakistan Agricultural Storage and Services Corporation) and Provincial food departments purchase about 20–25% of the wheat product (63). This wheat the purchased is milled and these products sold at discounted (subsidized) prices in a system of over 12,000 “Utility Stores” that have been established by the state-owned enterprise, Utility Stores Corporation (USC), which was created in 1971 (31).

This creates what political scientists would call a classic “distributive politics” system, where there are multiple overlapping constituencies with a vested interest in the continuation of this system (64). Big wheat farmers, mainly in rich Punjab province, have the luxury of assured government procurement at rates well above market prices. At the same time, urban consumers in politically sensitive cities such as Karachi and Lahore can access cheap wheat flour, which is vital to flatbread, the main source of calories for a large number of low-income households (65). Moreover, the milling and distribution chains afford additional opportunities for rent-seeking and patronage jobs in a way that supports solidity of the system within Pakistan's political economy (66).

The years 2008 to 2010 present a fascinating opportunity for the implementation of a practically-desired Pakistani subsidy regime as they were impacted by multiple, interrelated external shocks that hungered pieces in combination to create space for reforms. High food prices High international wheat prices during 2007–2008 reaching above $400/ton, from around $200, imposed significant fiscal pressure on Pakistan's subsidy program (67). This external pressure came at a time of domestic political change in the aftermath of 2008 elections and balance of payments crisis which had led to sharp decline in forex reserves. The Stand-By Arrangement with the International Monetary Fund (IMF) that was secured in November 2008 laid down a clear conditionality of decreasing food and fuel subsidies as part of securing financial assistance, thereby bringing about significant external pressure for reform, which was relatively removed from the prevailing domestic politics (68, 69).

The government made significant reforms in the wheat subsidy system in 2009 and 2010 through a reduction of about 30% on the procurement quantities, raising consumer prices at Utility Stores by 35 percent (in real terms), and introducing targeting mechanism for capping sales to lower income households (70). These reforms represented a substantial adverse shock to levels of subsidies and thus provided plausibly exogenous changes for analysis. The combination of price shocks and IMF conditionality buttresses a strong case for exogeneity, as the timing and degree of the reform were driven more by international than domestic politics (68, 71). The sequence of these external shocks and the consequent domestic policy responses is depicted in Figure 2.

**Figure 2:**
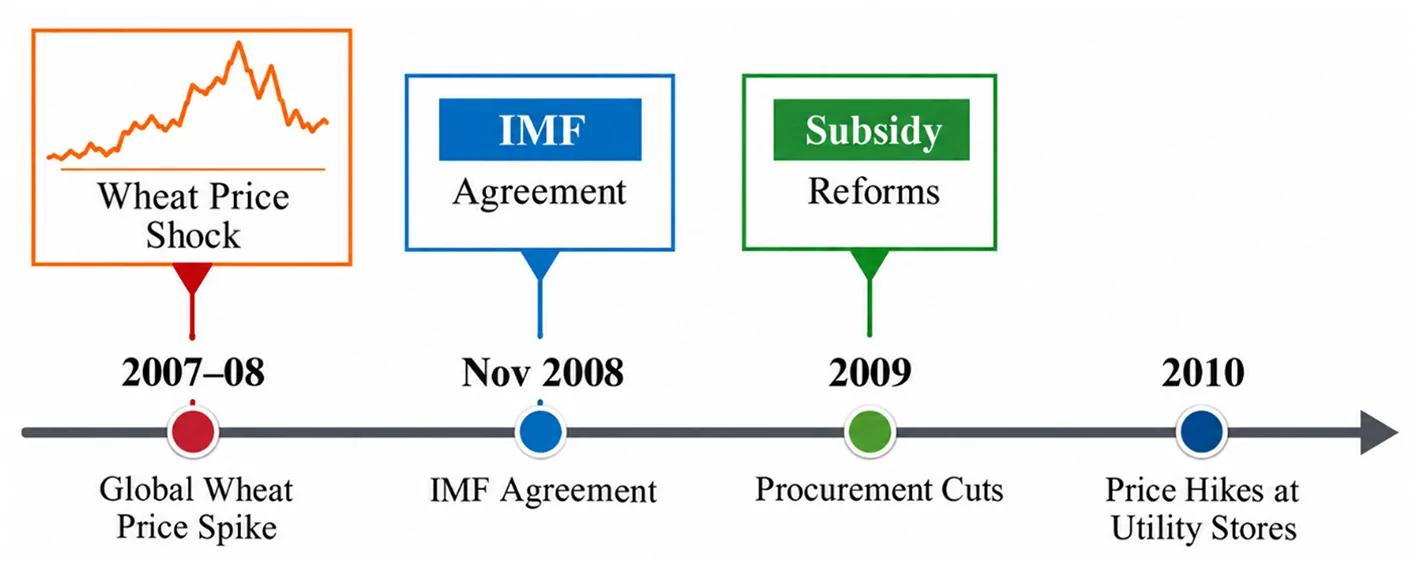
Timeline showing key events to Pakistan's wheat subsidy reforms.

Consistent with the literature (39, 72–75), this paper uses a conceptual framework to investigate how borrowing constraints and food price shocks interact to have long-term effects on child outcomes. When global food prices are high, the cut in subsidy on staple foods creates severe pressure for households without access to credit. These families fail to optimize their consumption, thus consuming less than the normative requirements in crucial stages of early life. Since nutrition during early childhood is a key determinant of later human capital accumulation, these temporary shocks are likely to have long-lasting effects. A simplified representation of these mechanisms is provided in Figure 3 panels (A–D) below.

**Figure 3:**
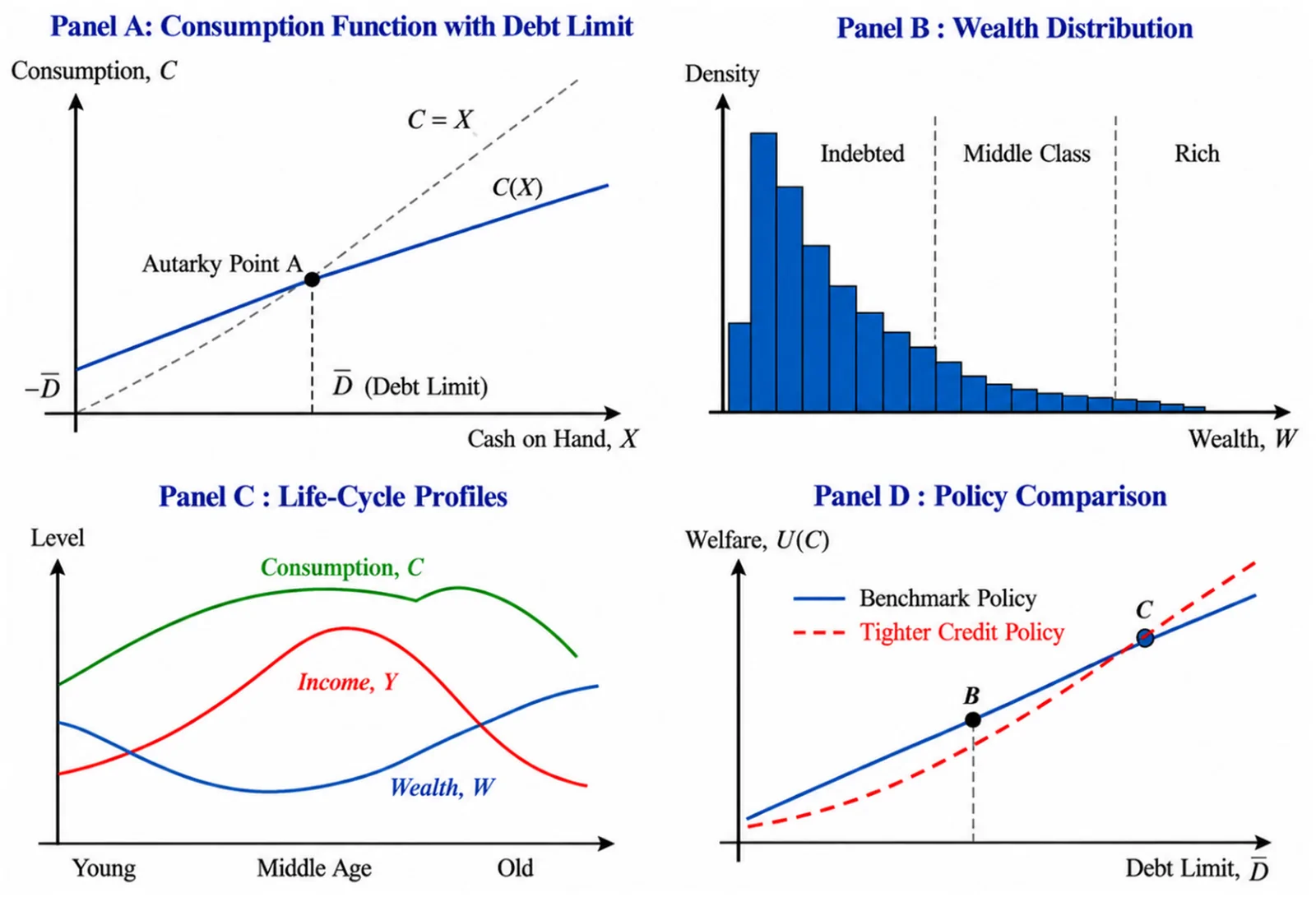
Conceptual framework—credit constraints, nutrition, and life-cycle outcomes.

The Figure 3 provides a stylized life-cycle framework that helps explain the economic channels through which food price shocks, credit constraints, and long-run child outcomes are connected. Not posited as a model in itself, such system becomes an illustrative device for conceptualizing the empirical relationship.

Four keys' elements are shown in Figures 3(A–D). Panel (a) is a consumption function under borrowing constraints, which shows that negative income shocks can cause very large decline in expenditure for families close to their debt ceiling. Panel (b) show evidence of a right-skewed wealth distribution, indicating that a significant fraction of households may be subject to these constraints. Panel (c) shows the life-cycle income, consumption and wealth paths, highlighting that infancy is particularly fragile in terms of child growth where adequate nutrition is crucial to Human Development. Last, Panel (d) compares welfare outcomes under alternative credit arrangements, showing that tighter borrowing constraints can worsen welfare implications of temporary shocks.

Overall, this model demonstrates how ephemeral disturbances to food prices can have long-term consequences, especially for households with limited access to credit and when a shock occurs during times of early household formation. The figure is for an illustrative purpose, and to help understand the empirical findings.

## 4 Methodology

### 4.1 Data bases and sample building

Empirically, this module relies on a wide range of databases and harmonizes them to construct an unbalanced repeated cross-sectional dataset at the child and district-levels. It uses three waves of the Pakistan Demographic and Health Survey (PDHS) data (2006–07, 2012–13 and 2017–18) (14). This dataset does not include information that tracks the same group of respondents over an extended period, making longitudinal analysis impossible. The study can only adopt a repeated cross-sectional cohort design to conduct empirical analysis, and all observed data are recorded at two levels: one is the district level, and the other is the cohort level. The so-called repeated cross-sectional cohort design refers to the overall plan for collecting and analyzing data during the research process, which collects and analyzes information from different research samples at different time points. The observed data used are also organized and aggregated according to these two levels: one set is district-level data organized by administrative region, and the other is cohort-level data organized by research subjects that share common characteristics within the same group. These surveys are standardized representative by national protocols, termed demographic and health survey (DHS), which systematically provide information for variables in the health, nutrition, and socio-economic domains of households. For these surveys, a Stratified Two-Stage Sampling design is implemented with Primary Sampling Units (PSUs) selected with Probability Proportional to Size and households systemically sampled with PSUs. The sample includes children 0 to 59 months old with complete anthropometric data and biologically plausible height-for-age z-scores (HAZ) (within the range recommended by World Health Organization, > −6 < +6) (76). This is followed by a fairly stringent selection procedure resulting in a final sample of 37,378 children from a total of 120 districts (76, 77).

Data on public distribution system (PDS) are obtained from the Annual Reports of the Utility Stores Corporation for 2005–07 and contain a complete list of all active outlets, including their date(s) in operation. Using geographic information from the 1998 Population Census, this study geocodes these store locations to their corresponding district boundaries. This matching requires that the administrative boundaries at the time of 1998 and the respective survey years (including all years up to 2006) are compared face by face, which is done using concordance tables created by Pakistan Bureau of Statistics for boundary matching (78).

Further, the district-level aggregates of Pakistan Standardized Achievement Tests (PSAT)—filled out by 5th and 8th grade students—capture educational outcomes on an annual basis from 2014 to 2019. Such assessments of student performance in these subjects, based on a common curriculum, permit national comparisons of achievement trends across districts and cohorts (79, 80). The test results can be linked to nutritional information by year of birth and testing years, and they are then used to demonstrate that shocks experienced in early childhood with respect to nutrition in turn have consequences for educational outcomes later on (81).

To further bolster robustness and context, additional data derived from Multiple Indicator Cluster Surveys (MICS) supplement child nutrition indicators; Household Integrated Economic Surveys (HIES) provide in-depth consumption expenditure information; provincial agriculture departments supply agricultural production statistics; and electoral data from the Election Commission of Pakistan assist in district matching and contextual analyses. Districtwide contextualization of electoral data. It must be noted that the Election Commission of Pakistan provided electoral data for some districts and few references are made to indicate those instances. Note, however, that these electoral data cannot be used as outcome measures in our main analysis due to a unit-of-analysis mismatch: the Election Commission data is disaggregated by constituency level, which does not map cleanly onto district boundaries in the PDHS and alternate years are incompatible with our cohort-based identification strategy. For this reason, we rely on the data only for descriptive contextual purposes and do not use them as dependent variables in our regression framework.

### 4.2 Variable construction

The main treatment variable measures exposure to the public wheat distribution system at the district level before reforms were implemented. Utility store density is calculated as the number of utility stores per 10,000 residents, based on 1998 census data. It relies on the idea that locations of stores opened before the reform period are persisting patterns of infrastructure, not responses to future policies. This study followed the previous studies in which the utility stores density index formula has been widely used (82, 83). This measure is then standardized to facilitate interpretation:


$$\begin{array}{l} z_{d}=\frac{x_{d}-\mu }{\sigma } \end{array}$$

*z*_*d*_ = is the standardized exposure for district “*d*”.
*x*_*d*_ = are the observed stores per 10,000 residents in district “*d*”.
μ = the mean store density across all districts.
σ = is the standard deviation of store density across all districts.

Table A1 in appendix examines the observable correlates of pre-reform store density. Store density is positively associated with population density and urban share but uncorrelated with baseline literacy rates or wheat production. This pattern reflects the idea that urbanization shapes where stores locate, but not through initial conditions in child nutrition—when district fixed effects are included. Nevertheless, we cannot rule out unobserved time-invariant confounding, a limitation we return to in the discussion.

In this study the detailed form of the variable is:


$$Exposure_{d}^{std}=\frac{Stores\;per\;10,000\;in\;district\;d-Stores\;per\;10,000}{\sigma (Stores\;per\;10,000)}$$

The timing variable identifies children born after the major reforms began implementation. Based on analysis of policy documents and parliamentary debates, defined as:


$$Post_{t}=\{\begin{array}{l} 1\;if\;childs\;birth\;year\;\geq 2009\\ 0\;if\;childs\;birth\;(otherwise) \end{array}$$

This definition identifies the period when savings in costs of procurement and higher consumer prices were fully brought to bear, with sensitivity analyses performed at alternative base years cut-off.

The main dependent variable is the height for age z-score (HAZ) using WHO child growth standards (84). The height-for-age z-score (HAZ) is the height of a child expressed as standard deviation scores compared with mean heights for children of a similar age and sex in healthy populations. Stunting is operationally defined as HAZ < −2, which means moderate to severe linear growth retardation.

Secondary outcomes include dietary diversity scores calculated based on 24-h recalls, following the WHO Infant and Young Child Feeding (84). Further, each testing year's educational test scores are standardized with variable mean 0 and standard deviation 1. Indices of women's empowerment are compiled from the Pakistan Demographic & Health Survey (PDHS) model questionnaires that measure autonomy in decision-making, freedom of mobility, attitudes toward gender-based violence and exposure to media (85).

Control variables are selected based on established determinants of child nutrition and human capital formation (23, 32). Child-level controls include age in months, sex, and birth order. Maternal controls include age, education in completed years, and height in centimeters. Household controls include a wealth index constructed from principal components analysis of asset ownership and housing characteristics (86), urban/rural location, and province fixed effects. District-level controls include population density, adult literacy rate from the 1998 census, and wheat production per capita. Descriptive statistics for all primary variables are presented in Table 1.

**Table 1:** Summary statistics.

Variable	Mean	SD	Min	Max	Observations
Panel A: child-level variables					
Height-for-age z-score	−1.48	1.62	−6.00	5.00	37,378
Stunting (HAZ < −2)	0.368	0.482	0	1	37,378
Age (months)	29.4	17.2	0	59	37,378
Female	0.483	0.500	0	1	37,378
Birth order	3.2	2.1	1	15	37,378
Mother's education (years)	4.2	4.8	0	18	37,378
Mother's height (cm)	152.1	6.3	130	180	37,378
Wealth index (1–5)	2.9	1.4	1	5	37,378
Urban residence	0.324	0.468	0	1	37,378
Panel B: district-level variables					
Utility stores per 10,000 population	0.42	0.31	0.02	1.45	120
Exposure (standardized)	0.00	1.00	−1.28	3.21	120
Population density (persons/km^2^)	456	812	12	6,432	120
Adult literacy rate (%)	45.2	12.8	18.3	72.1	120
Wheat production (tons per capita)	0.12	0.08	0.01	0.42	120

Source: PDHS 2006-2018, Utility Stores Corporation, Population Census 1998.

### 4.3 Analytical approach

The main empirical strategy is a difference-in-differences framework that exploits both time series variation from the reforms between 2008-2010, and cross-sectional district-level variation in exposure to the public distribution system. This design is consistent with previous Indian studies evaluating public distribution reforms (87, 88). The baseline specification can be estimated as:


$$HAZ_{idt}=\alpha +\beta \;(Post_{t}\times Exposure_{d}^{std}+\gamma x_{idt}+\delta_{d}+\eta_{t}+\epsilon_{idt}$$ (1)

where is the height-for-age z-score *HAZ*_*idt*_ of a child “*i”* in district “*d”* born in year “*t”* is a vector of controls *x*_*idt*_, and are district δ_*d*_ and birth-year η_*t*_ fixed effects respectively, and finally, is the error term ϵ_*idt*_ clustered at the district level to account for possible serial correlation. Coefficient “β” captures the reform's differential impact on child nutrition in high- versus low-exposure districts. The identifying assumption—that unobserved time-varying district characteristics are uncorrelated with pre-reform utility store density, conditional on fixed effects and covariates—is supported by event-study evidence showing no pre-trends (joint *F*-test *p* = 0.42). Because store density proxies' treatment intensity rather than actual household subsidy reductions, the estimate represents an intention-to-treat effect.

Since the parallel trends assumption that is fundamental for Causal Inference, we check this antecedent by estimating an event study specification as:


$$\begin{array}{l} HAZ_{idt}=\sum_{k=-4}^{5}\beta_{k}(1\left\{t=2004+k\right\}\times Exposure_{d}^{std}+\gamma x_{idt}+\delta_{d}\\ \;+\eta_{t}+\epsilon_{idt} \end{array}$$ (2)

The coefficients β_*k*_ test for pre-trends *k* < 0, while the coefficients for *k*≥0 produce an estimate of how treatment effects develop over time. Rejection of parallel trends is not achieved using a joint F-test on pre-period coefficients (*p* = 0.42).

The event study specification given in Equation (2) uses the year of a child's birth as its time unit, the event-study specification uses 2004 as the omitted reference birth cohort. The first full year following implementation of the wheat-subsidy reforms. Pre-reform coefficients (k < 0) test for differing nutritional trajectories between high and low exposure districts. The joint *F*-test (*p* = 0.42) supports the parallel trends assumption, despite limited power. Appendix robustness checks using district-specific linear trends yield consistent results. We define pre-reform birth cohorts (2005–2008) using data from the 2006–07 and 2012–13 waves of the survey, since all children in this period would have born before the reform was initiated. Post-reform cohorts (2009–14) are sampled from the 2012–13 and 2017–18 waves. This design yields a maximum of four pre-reform birth cohorts and six post-reform birth cohorts. Importantly, identification comes from the variation in exposure intensity across districts within cohort years and not direct comparisons across survey waves. The coefficient for pre-reform (k < 0) tests whether districts with high vs. low exposure had significantly different nutritional trajectories in the absence of reforms.

Equation (1) was estimated separately by household wealth quintile for heterogeneity, child age at exposure (0–23 months vs. 24–59 months), urban residence, rural residence and province for exploring heterogeneous treatment effects. Tests of coefficient equality are used in order to make inference regarding the varied effects across subgroups determined by vulnerability and contextual factors (89).

In order to study the medium-run educational effects, we construct a district-cohort level measure of nutritional shocks and relate it to test scores.


$$\begin{array}{c} TestScore_{dc}=a+\bar{\theta {HAZ\;Shock}_{dc}}+\emptyset {\;z}_{dc}+\delta_{d}+\eta_{c}+u_{dc} \end{array}$$ (3)

where $\bar{{HAZ\;Shock}_{dc}}\;$ is the mean residual HAZ for cohort “c” in district “d” in Equation (1), representing that part of child nutritional status related to exposure to reforms. *z*_*dc*_ includes district-cohort controls (e.g., teacher-student ratios, school infrastructure).

For political attitudes and behaviors, I estimate specifications similar to Equation (1) using women's empowerment indices as dependent variables. Given data limitations on direct political behavior, these indices serve as proxies for civic capacity.

We conduct several robustness checks to reduce the likelihood of potential identification threats: (1) Placebo tests – we assign treatment to either 2005 or 2006 and investigate if effects are observed spurious; (2) Alternative exposure measures - wheat production on a per capita basis at the district level and distance to the nearest flour mill, thus providing alternative operationalizations of intensity of treatment; (3) Sibling fixed effects models - outcomes for children born in districts before and after reforms are compared, controlling for unobserved maternal characteristics. While the sibling fixed-effects sample includes 8,234 children from 3,427 mothers (1,856 have children born across the reform threshold). This subsample does not differ systematically from the full sample in household wealth (*p* = 0.34) or maternal education (*p* = 0.28); (4) Migration controls - concerns regarding residential mobility, can be addressed using mother's district of birth as an alternative geographic identifier; and (5) Controls for concurrent shocks through measuring variations in rainfall, temperature, and conflict events at the district-year level.

A methodological note on educational achievement analysis (Equation 3). The HAZ shock variable *HAZ Shock*_*dc*_ is based on the district-cohort mean residuals from of Equation (1). Since this variable is a generated regressor, estimated in a first stage and subsequently used as an explanatory variable in a regression given by Equation (3), the standard errors calculated for this bundle require adjustments to account for the estimation uncertainty carried forward from the first stage (90, 91). We account for this by reporting second-stage inference using bootstrapped standard errors (500 replications), which involves re-estimating the first stage for each bootstrap iteration and propagating the first-stage uncertainty into the second-stage estimation. This is a conservative approach and with this choice, the standard errors reported take into account the entire estimation process. However, the educational outcomes are still correlational - we caution that they should be read as more suggestive evidence of enduring associations rather than causal estimates.

## 5 Results

Our analysis divides the evidence into three tiers, each with a different level of reliability. The first tier uses the difference-in-differences method to conduct estimation, yielding highly persuasive quasi-experimental evidence that can link exposure to the reform to children's nutritional status. However, for this conclusion to hold, the parallel trends assumption for different groups must first be satisfied. In simple terms, only when the prerequisite conditions required by this method are met can this association conclusion be valid. The household dietary adjustments observed in the second tier can support the logical validity of the nutritional mechanism: that is, the transmission path through which the reform indeed acts on children by influencing their nutritional status is logically sound. The estimations targeting educational attainment and civic competence in the third tier are suggestive associative evidence, which can link the abrupt change in nutritional status to various subsequent developmental outcomes. The reliability of this type of evidence is inferior to that of the first two tiers; it can only suggest that a correlation exists between the two, and cannot draw a definitive causal conclusion.

### 5.1 Main effects on child nutrition

The empirical analysis shows that the decline in wheat subsidies resulting from the 2008–2010 reforms had significant negative effects on child nutritional status, particularly in districts with high pre-reform reliance on the public distribution system. The estimates from simple difference-in-differences that are presented in Table 2 indicates a reduction of height-for-age z-scores by about 0.28 standard deviations among children born on average after the reforms in high-exposure districts, relative to their counterparts in low-exposure ones. This effect is statistically significant at the 1% level and has economically relevant implications: The estimate represents a substantial shift in children's height-for-age relative to the WHO reference distribution.

**Table 2:** Impact of subsidy reforms on child height-for-age (Dependent variable: height-for-age z-score).

Specification	(1)	(2)	(3)	(4)
Post × Exposure	−0.283^***^	−0.261^***^	−0.374^***^	−0.247^**^
	(0.085)	(0.082)	(0.101)	(0.094)
Post	−0.102	−0.088	−0.121	−0.095
	(0.064)	(0.061)	(0.072)	(0.067)
Controls:				
Child characteristics	No	Yes	Yes	Yes
Mother characteristics	No	Yes	Yes	Yes
Household wealth	No	Yes	Yes	Yes
District FE	Yes	Yes	Yes	Yes
Year FE	Yes	Yes	Yes	Yes
Sample	Full	Full	Poorest 40%	Sibling FE
Observations	37,378	37,378	14,951	8,234
R-squared	0.187	0.243	0.251	0.312

^***^p < 0.01, ^*^p < 0.05, *p* < 0.10. Robust standard errors clustered at district level in parentheses. Child characteristics include age, sex, and birth order. Mother characteristics include age, education, and height. Column 4 includes mother fixed effects and uses only households with multiple children surveyed. All specifications include district and birth-year fixed effects.

Column 1 presents the basic difference-in-differences estimate without controls: a one standard deviation increase in pre-reform exposure is associated with a 0.283 standard deviation decline in HAZ scores after the reforms. Column 2 adds controls for child, mother, and household characteristics, reducing the estimate slightly to −0.261 but maintaining statistical significance at the 1% level. Column 3 restricts the sample to households located in the bottom 40% of the wealth distribution. Here we observe that the coefficient increases to −0.374 which shows that for poor households, there is evidence of more depress impact. This is consistent with their greater reliance on affordable basic foods and the more limited opportunities they have to change their diets. Column 4 uses a sibling fixed effects model, that allows us to compare children who are born to the same mother and with different birth years before and after the reforms. The estimated coefficient of −0.247 in the sibling fixed-effects specification is very close to the main specification estimate indicating that the observed incidence is not due to time-invariant characteristics of a household. Given a baseline mean HAZ of −1.48 (SD 1.62), the estimated 0.28 SD decline represents approximately a 1.0 cm height deficit by age five. This effect size matches the 0.25–0.35 SD declines reported in other major nutritional shocks (93).

The event study analysis is consistent with the parallel trend's assumption necessary for a valid causal interpretation. The estimates of the event study coefficients are presented in Table 3. The pre-reform coefficients are individually and jointly insignificant (*p* = 0.42), showing no evidence of differential pre-trends between high- and low-exposure districts. This is consistent with the identification assumption that, in the absence of the reforms, child nutrition outcomes would have evolved similarly across districts with varying exposure levels to this public distribution system.

**Table 3:** Event study of reform effects on child height-for-age (Dependent variable: height-for-age z-score).

Year relative to reform	Year	Co-efficient	Standard error	*p*-value
−4	2005	0.012	(0.045)	0.789
−3	2006	−0.021	(0.038)	0.580
−2	2007	0.008	(0.042)	0.849
−1	2008	−0.015	(0.039)	0.698
0	2009	−0.142	(0.057)	0.013
1	2010	−0.201	(0.062)	0.001
2	2011	−0.258	(0.071)	0.000
3	2012	−0.294	(0.082)	0.000
4	2013	−0.312	(0.089)	0.001
5	2014	−0.328	(0.094)	0.001

^***^p < 0.01, ^**^p < 0.05, *p* < 0.10. Bold values indicate the reform year (2009), the first year in which the reform effect becomes statistically significant. Estimates from the event study specification (Equation 2) with full controls. The reference year is 2004. Standard errors are clustered at the district level. For the pre-period coefficients, a joint F-test fails to reject the null hypothesis of parallel pre-trends (*p* = 0.42).

The post-reform coefficients turn negative and are statistically significant, becoming larger in absolute values across time periods. This timing pattern also implies the presence of cumulative effects, since children experienced prolonged exposure to lower subsidies. Such exposure could have resulted in continuing dietary changes or developmental delays, which contribute to an overall burden of deficits that accumulate during the vulnerable period of early childhood. The results specified in Table 3 are depicted visually using the event study coefficients and are shown in Figure 4.

**Figure 4:**
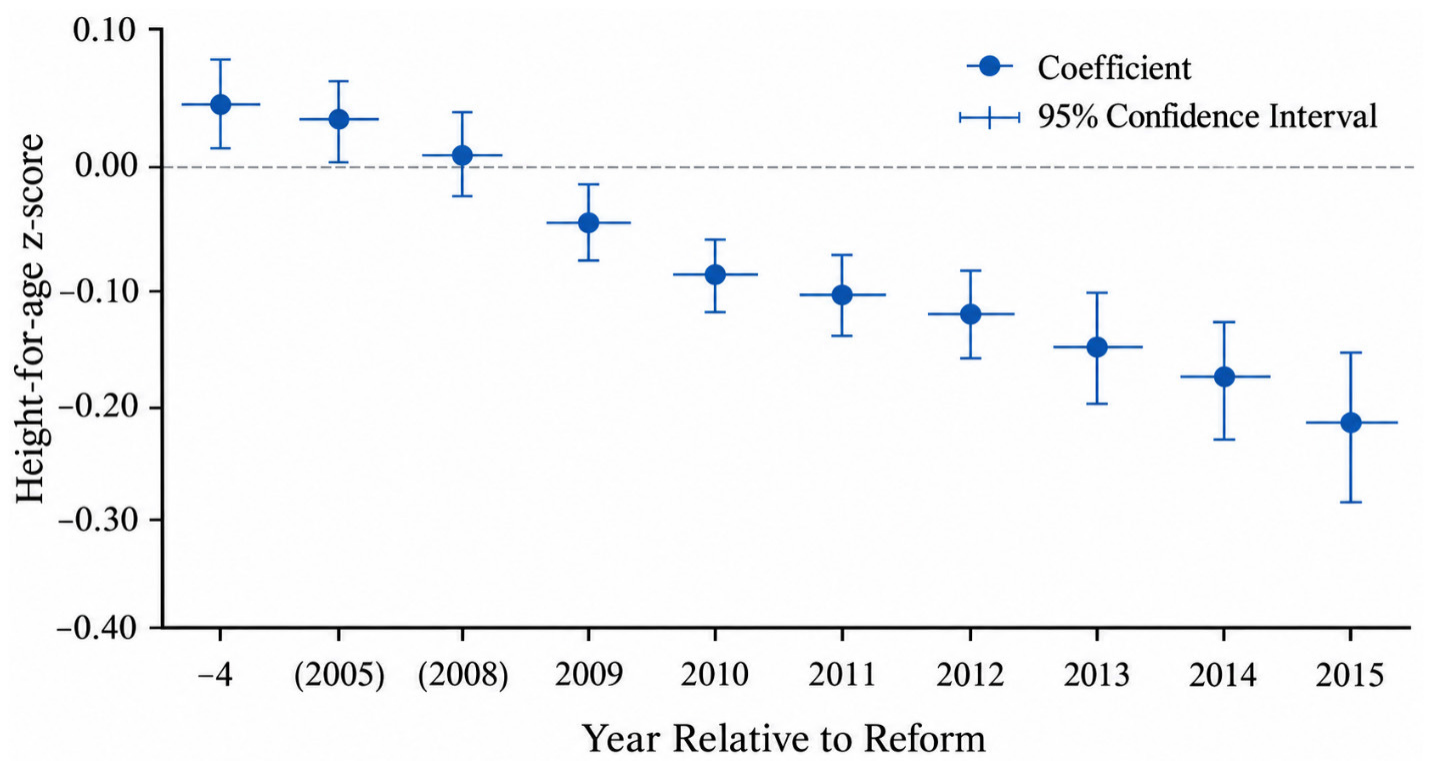
Visual representation of Table 3 coefficients with confidence intervals.

### 5.2 Heterogeneous effects

The analysis highlights large heterogeneity in treatment effects across subgroups of the population, providing valuable information on which children were more vulnerable to subsidy cuts. These heterogeneous effects are demonstrated in Table 4, where we show three of the more salient patterns that map well onto theory predicting vulnerability and critical periods of development.

**Table 4:** Heterogeneous treatment effects by subgroup.

Subgroup	Coefficient (post × exposure)	Observations	*p*-value for subgroup difference
By child age at measurement			0.032
0–23 months	−0.338^***^	15,672	
	(0.102)		
24–59 months	−0.198^**^	21,706	
	(0.087)		
By household wealth quintile			0.008
Poorest 20%	−0.401^***^	7,476	
	(0.118)		
Middle 60%	−0.248^***^	22,427	
	(0.089)		
Richest 20%	−0.142	7,475	
	(0.101)		
By Urban/Rural residence			0.182
Urban	−0.231^**^	12,113	
	(0.098)		
Rural	−0.276^***^	25,265	
	(0.085)		
By province			0.024
Punjab	−0.312^***^	22,451	
	(0.095)		
Sindh	−0.186^*^	8,342	
	(0.101)		
Khyber Pakhtunkhwa	−0.142	4,892	
	(0.124)		
Balochistan	−0.401^***^†	1,693	
	(0.142)		

^***^p < 0.01, ^*^p < 0.05, *p* < 0.10. All specifications include full controls and district and year fixed effects. Standard errors clustered at district level in parentheses. Subgroup differences p-value: tests if the treatment effect is different per category. †: The estimate for Balochistan is based on 1,693 observations from 14 districts. With a low number of clusters, standard errors clustered at the district level may be too small. This is why this estimate should be taken cautiously.

The effect is significantly larger for children exposed at ages 0–23 months than at 24–59 months (−0.338 vs. −0.198 SD, *p* = 0.032), reflecting early childhood's greater biological sensitivity to nutritional shocks. This age gradient is consistent with years of life lost attributable to malnutrition and critical windows for linear growth reported by the medical literature, indicating that reforms particularly benefitted children in their first 1,000 days of life. The treatment effects exhibits pronounced wealth gradient in the second result. The greatest difference occurred for the poorest 20% of households (−0.401 SD, *p* < 0.01) and, conversely, there was no significant effect in the richest 20% (−0.142, *P* = 0.16). This pattern is observed according to the interpretation of poorer households having higher cost share and lower substitution possibilities as well as inability to absorb food price increases suffer more from subsidy cuts. In line with the aforementioned results, nutritional costs associated with the reform are highly regressive; we find a difference between q1 and q5 (*p* = 0.008). Third, regional inequalities produce the most robust effects in Punjab (−0.312) and Balochistan (−0.401), average effects in Sindh (−0.186), and insignificant impacts in Khyber Pakhtunkhwa. These heterogeneities clearly account for differences in relative importance of wheat as part of the diet, ease of sourcing substitutes to wheat and local level implementation of reforms. The differential impact of the subsidy shock across wealth quintiles is reflected in Figure 5.

**Figure 5:**
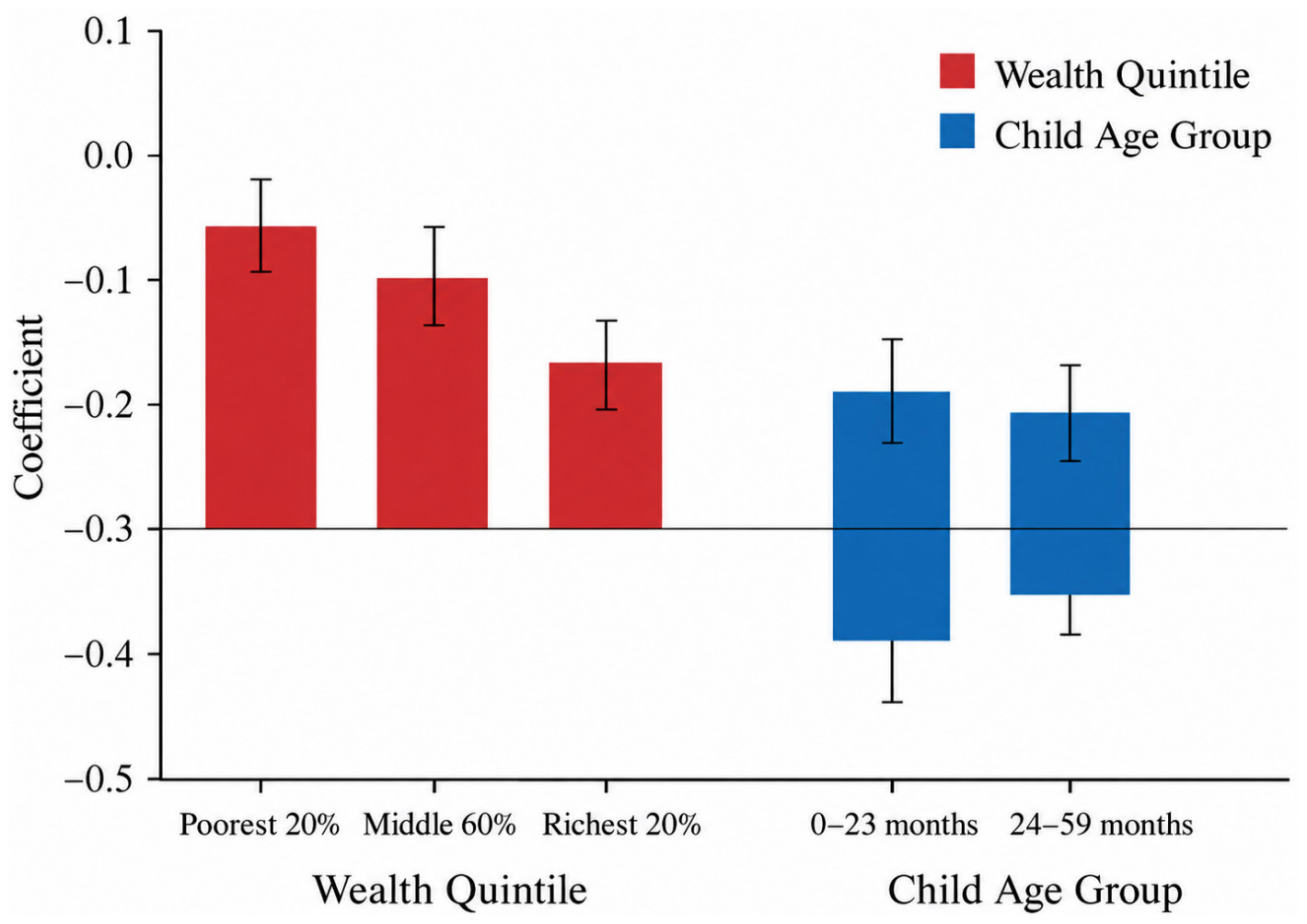
Bar chart comparing coefficients across wealth quintiles and age groups (heterogeneous impact of wealth).

### 5.3 Mechanisms: dietary changes

Analysis of household dietary patterns provides supporting evidence consistent with a nutritional mechanism rather than a formal mediation analysis. Table 5 indicates the estimates of change in various dietary indicators by exposure as a percentage relative to counterfactual values, indicating an additive trend toward metabolic disease among districts with highest reform exposure.

**Table 5:** Impact on household dietary patterns (Dependent variables: various dietary measures).

Outcome	Coefficient (post × exposure)	Observations	Mean in control
Dietary diversity score (0–7)	−0.42^***^	18,245	3.21
	(0.12)		
Days consumed pulses (past week)	−0.58^**^	18,245	2.84
	(0.23)		
Days consumed dairy (past week)	−0.61^***^	18,245	4.12
	(0.19)		
Days consumed meat/fish (past week)	−0.23	18,245	1.76
	(0.18)		
Days consumed fruits (past week)	−0.37^*^	18,245	2.45
	(0.20)		
Days consumed vegetables (past week)	−0.44^**^	18,245	4.89
	(0.21)		
Total food expenditure (log)	−0.08^*^	12,672	9.42
	(0.04)		
Wheat flour expenditure share	0.06^**^	12,672	0.18
	(0.03)		

^***^p < 0.01, ^*^p < 0.05, *p* < 0.10. Dietary data from PDHS 2012-13 and 2017-18. Expenditure data from HIES 2010–11 and 2015–16. All specifications include full controls and fixed effects. Standard errors clustered at district level in parentheses.

Households in high-exposure districts experienced significant declines in dietary diversity following the reforms, with particular reductions in consumption of protein-rich foods (pulses, dairy) and fruits and vegetables. Despite these declines in food quality, wheat flour's share in food expenditure increased, suggesting that households-maintained calorie consumption by prioritizing the (now relatively more expensive) staple over more nutritious foods. The patterns of these changes we have observed are completely consistent with a long-standing prediction in theoretical circles — that prediction states that if commodity prices fluctuate, households will adjust the categories of consumer goods they purchase when shopping, and will no longer only stick to the few items they used to buy regularly. These consistent observational results provide support for the transmission pathway of nutritional changes, but they cannot fully confirm that this mechanism of action is indeed valid.

### 5.4 Medium-term educational consequences

Nutritional shocks in early childhood have long-term effects on education. Table 6 reports estimate of the relationship between district-cohort level nutritional shocks and math scores in later standardized assessments. Results reveal consistently negative correlations that are significant after controlling for educational inputs and district specific characteristics.

**Table 6:** Nutritional shocks and educational achievement (Dependent variable: standardized mathematics test score).

Specification	(1)	(2)	(3)	(4)
HAZ shock (age 0–2)	−0.224^***^	−0.208^***^	−0.231^***^	−0.219^***^
	(0.079)	(0.076)	(0.082)	(0.079)
HAZ shock (age 3–5)	−0.118^*^	−0.102	−0.125^*^	−0.117^*^
	(0.073)	(0.071)	(0.076)	(0.074)
Controls:				
District characteristics	No	Yes	Yes	Yes
School infrastructure	No	No	Yes	Yes
Teacher qualifications	No	No	No	Yes
District FE	Yes	Yes	Yes	Yes
Cohort FE	Yes	Yes	Yes	Yes
Observations	840	840	840	840
R-squared	0.412	0.438	0.451	0.463

^***^p < 0.01, ^**^p < 0.05, ^*^p < 0.10 The unit of observation is the district-cohort, where each cohort corresponds to a year of birth (*N* = 840 district-cohorts). HAZ shock The HAZ shock variable is the average of the residual Height-for-Age Z-score from Equation (1), averaged at the district-cohort level. To adjust for uncertainty in the second stage due to using first-stage residuals as a regressor, we present bootstrapped standard errors (500 replications) that re-estimate the first stage per bootstrap iteration. In this way, the estimation uncertainty from the first stage is carried into the second-stage standard errors. Standard errors are reported in parentheses, and they are clustered at the district level. After the bootstrap correction, coefficients remain robust and statistically significant in their association with education.

This means that for each one-standard-deviation decrease in HAZ between the ages of 0–2, adolescent maths test scores decline by 0.22 SDs. For ages 3–5, the corresponding effect is also relatively small (−0.12 SD) and only marginally significant. These findings reinforce other research showing the transgenerational effect of early childhood nutritional deficits on cognition (43, 102). Controlling for district-level school infrastructure, teacher qualifications, and education expenditure (columns 2–4), in any particular year does not change the estimates which indicates that the correlation is mainly reflective of early childhood inputs rather than contemporaneous differences in educational inputs. We do caution that because the HAZ shock is a generated regressor carrying first-stage estimation error, these results should be interpreted as suggestive evidence of long-term associations rather than causal estimates of an education channel. The bootstrap-corrected standard errors shown in Table 6 echo those obtained from the generated regressor correction indicating that no statistical significance is lost when accounting correctly for these associations.

Figure 6 additionally shows that those nutritional shock indicators experienced in early childhood predict weaker scores on mathematics tests at the adolescent age. The effects of the nutritional shocks that arise at ages 0–2 are stronger than those observed between the ages of 3 and 5, which is consistent with estimates reported in Table 6. These conclusions can only prove relevant long-term correlations, and cannot yield causal estimates for educational pathways. That is, we can only confirm that these factors have consistently co-occurred over the long term, and cannot establish the causal relationship that “following this educational impact pathway will definitely produce a specific outcome.” We can only calculate that correlations exist between each other, and cannot accurately sort out which is the cause and which is the effect. The reason for this limitation is that the HAZ shock variable used in the study is an artificially generated regression variable, which lacks the identification capability of a quasi-experiment—the type of capability that is required to determine major nutrition-related outcomes. In other words, HAZ shock is not a naturally occurring experimental variable that can help us clarify causal logics; it is constructed by researchers solely for regression analysis. It cannot, like a qualified quasi-experiment, accurately pinpoint which factors have produced those core nutrition-related outcomes, and naturally cannot derive rigorous causal conclusions.

**Figure 6:**
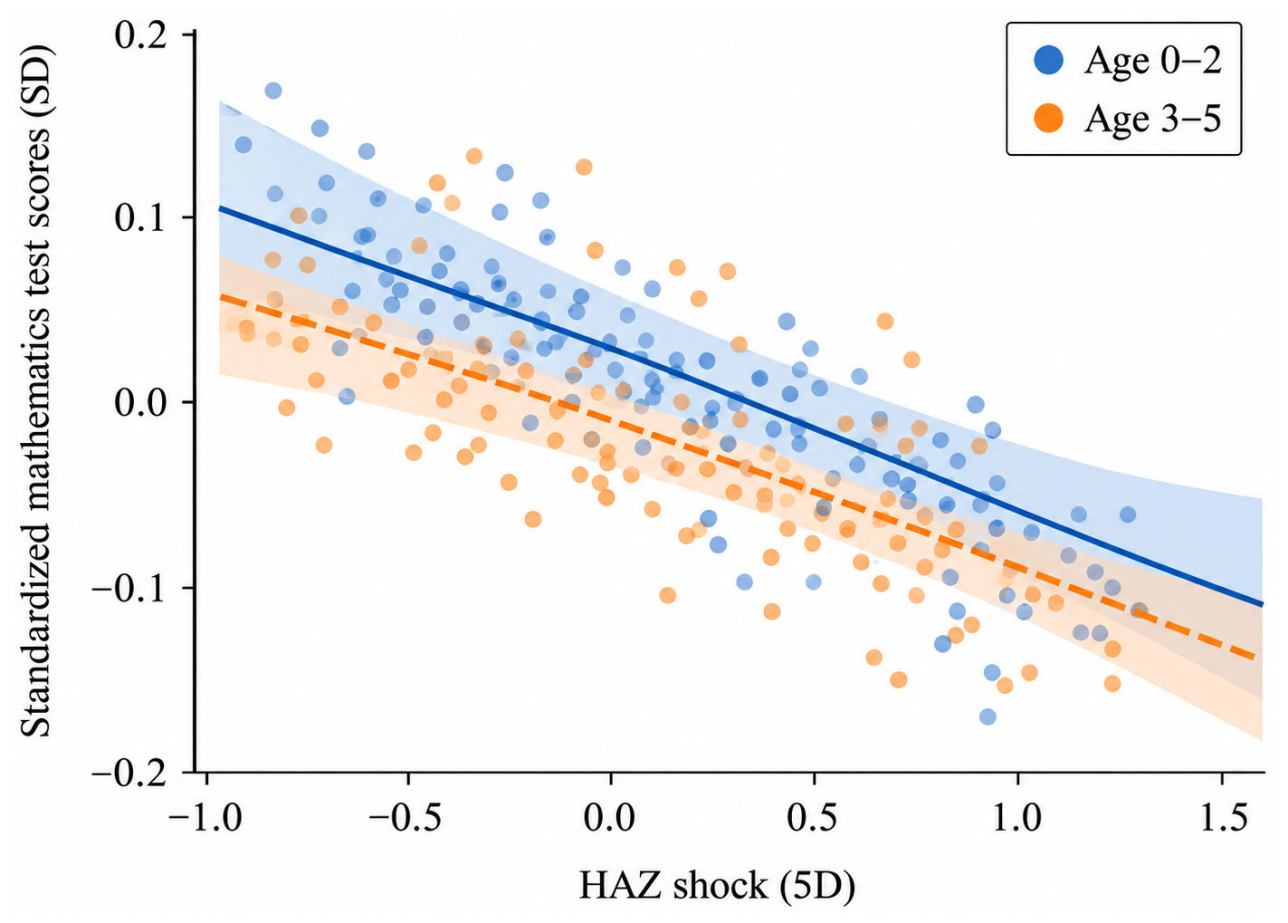
Nutritional shock vs. test scores.

### 5.5 Political attitudes and civic engagement

It is crucial to clarify before presenting the results, what our dependent variables have in common with political accountability. The women's empowerment indices from PDHS included multiple aspects such as intra household decision making autonomy, freedom of movement, rejection of gender-based violence, exposure to mass media and participation in community activities. These indices do not attempt to quantify political activities such as voting or signing petitions. However, in the case of Pakistan, there are three main reasons why these indicators theoretically matter to civic capacity. This is driven first and foremost by the access to political information through media coverage of elite performance. The second is that freedom of movement can be important for attending community meetings and taking action collectively, as well as interacting with local governance institutions. The third pathway links the rejection of hierarchical authority (and by extension societal attitudes toward gender-based violence) to how one views political accountability and willingness to challenge elite corruption. Accordingly, we regard these variables as proxies for civic capacity rather than measures of political accountability. Suggestive evidence is the result of similar patterns associated with multiple proxies; estimates from individual coefficients should be interpreted very tentatively.

Table 7 presents the estimated associations between the levels of pre-reform exposure and five standardized measures of women's empowerment. All five measures decrease in the most exposed districts: decision-making autonomy decreases by 0.134 SD; mobility freedom decreases by 0.182 SD; rejection of gender-based violence decreases by 0.211 SD; media exposure decreases by 0.087 SD; and community participation decreases by 0.156 SD. Based on these findings, it appears that women living in areas with higher levels of exposure report having less autonomy, less mobility, less media access, and less community involvement.

**Table 7:** Nutritional exposure and women's empowerment Condition (Dependent variables: standardized indices).

Outcome	Coefficient (post × exposure)	Observations	Interpretation
Decision-making autonomy	−0.134^**^	45,672	0.134 SD lower
	(0.062)		
Mobility freedom	−0.182^***^	45,672	0.182 SD lower
	(0.054)		
Rejects gender-based violence	−0.211^***^	45,672	0.211 SD lower
	(0.058)		
Regular media exposure	−0.087^*^	45,672	0.087 SD lower
	(0.049)		
Community participation	−0.156^***^	45,672	0.156 SD lower
	(0.051)		

^***^p < 0.01, ^*^p < 0.05, *p* < 0.10. All outcome indicators for the research have been uniformly converted into standardized indices. The coefficients listed in the table are used to measure the standard deviation difference corresponding to the Post × Exposure interaction term. The standard errors used to calculate the coefficients have been clustered by administrative district level.

The indices used here to measure civic capacity are better viewed as indirect measures of civic capacity than as direct indicators of civic behavior, as the indices were developed based on indirect indicators of civic behavior (i.e., not based on observation of whether people voted or signed petitions). There are three ways in which the indirect indicators relate to civic capacity: media coverage allows individuals to learn about how well leaders perform; freedom to travel enables individuals to attend community meetings and interact with local governmental agencies; and perceptions of hierarchical authority may reflect an individual's view regarding accountability. As such, Table 7 contains estimates that provide strong support for the civic capacity channel, but do not provide estimates of actual civic behavior.

In high-exposure districts, women report significant losses of autonomy in decisions related to the household and their mobility — increasing tolerance of violence by partners as well as less media exposure and less participation in community activities. However, while almost none of this research tracks political activity behaviors directly and all these indicators are best viewed holistically, collectively they tell a clear story: public space is increasingly becoming less the domain or welcome ambiance for citizens, and even the structures of authority around feminism itself appear to be shifting. This pattern closely maps onto theoretical expectations that economic hardship is expected to depress civic engagement.

### 5.6 Robustness and alternative explanations

The estimated effects are also robust to a large number of specification checks and alternative explanations. In the first place, placebo tests that incorrectly code the treatment year as 2005 or 2006 yield coefficients close to zero and statistically indistinguishable from zero (0.012 and −0.008 with standard errors of 0.045 and 0.039, respectively). This finding is consistent with the identifying assumption that, in the absence of reform, districts with both high and low exposure would have followed a common trajectory over time for nutritional outcomes.

Second, the quantitative results are consistent when alternative measures of exposure to the wheat distribution system are used. Wheat production per capita yields −0.228^***^ (0.077); distance to nearest flour mill (where shorter distance is higher exposure) yields 0.241^***^ (0.073); and historical wheat suitability results in −0.267^***^ (0.083). These results affirm that our conclusions are not sensitive to the particular metric selected to define subsidy dependence.

Third, in order to evaluate whether the results might be produced through other mechanisms than nutritional lock-in, Table 8 shows difference-in-differences estimates for four plausible alternative channels: general income effects, healthcare access, fertility responses, and migration. Each specification follows the structure of Equation (1), replacing the dependent variable with the alternative outcome listed in Table 8. In all cases, these coefficients do not attain statistical significance and are economically insignificant.

**Table 8:** Testing alternative mechanisms (Alternative channels for the effects of subsidy reforms).

Mechanism tested	Outcome variable	Coefficient (post × exposure)	Conclusion
General income effect	Household asset index (standardized)	0.02 (0.04)	No significant effect
Healthcare access	Full vaccination rate (children 12–23 months)	−0.01 (0.03)	No significant effect
Fertility response	Birth spacing (months since previous birth)	0.05 (0.06)	No significant effect
Migration	Share of mothers who are in-migrants	−0.003 (0.02)	No significant effect

Each row reports the difference-in-differences coefficient (β) from a separate regression of the form $Y_{idt}=\alpha +\beta \;(Post_{t}\times Exposure_{d}^{std}+\gamma x_{idt}+\delta_{d}+\eta_{t}+\epsilon_{idt}\;$ where the dependent variable *Y*_*idt*_ is the outcome listed in the second column. All specifications include the same set of child-, mother-, and household-level controls as in Table 2, as well as district and birth-year fixed effects. Robust standard errors clustered at the district level are shown in parentheses. The vaccination analysis is restricted to children aged 12–23 months. None of the coefficients are statistically significant at conventional levels (all *P* < 0.10).

Consistent with Table 8, supplemental analysis reveals that the reforms did not produce broader income shocks: we observe no significant effects on asset ownership or housing quality, and only a modest decline in total food expenditure (−0.08 log points). On the other hand, the change in dietary composition was large (Table 5), which corresponded to the one observed when substitution effects due to relative price changes are more pronounced as compared with an overall contraction in income. Health service utilization—vaccination coverage, antenatal care, disease prevalence—did not reveal any differential trend between high and low exposed districts. Fertility effects were very small: there were no detectable changes in birth intervals, parity progressions or maternal ages at births. In addition, the use of the mother's district of birth as an alternative measure of location generates an estimate that is very close to the preferred one (−0.247^**^ with SE 0.094), and migration flows do not show any systematic response to the reform.

Taken both together, these robustness checks support the conclusion that the effects we observe mainly operate through the nutritional channel identified in our conceptual framework, rather than via other economic, health, demographic or geographic transmission channels.

## 6 Discussion

These findings tie in with a number of strands of current political economy, development economics and human capital formation literature. Using evidence from Pakistan's wheat subsidy reforms, this article provides an empirical test of the nutritional lock-in hypothesis through its impact on child nutrition, education outcomes and civic participation. The implications of these findings are significant for understanding the interweaving of food policies with development trajectories and national political institutions, while we caution that the complete feedback loop from nutrition to political accountability and policy persistence remains an implication rather than a conclusion in this paper.

Specifically, an identification concern that we highlight before discussing the implications. The reform era also witnessed the onset of greater macroeconomic instability, which was characterized by shocks to international food prices and IMF intervention. High-exposure districts may well have differed systematically in their exposure to other contemporaneous shocks compared with low-exposure districts. There are multiple reasons to believe that this is not an issue: district fixed effects absorb time-invariant differences; the event study shows no differential pre-trends; and the sibling fixed effects specification (Table 2, column 4) controls for unobserved household-level characteristics. Yet we cannot entirely exclude that high-exposure districts had heterogeneous responses to macroeconomic circumstances during the reform era. The robustness checks suggest that this assumption is plausible, but we would not consider it possible to test the hypothesis that all differential responses during these settings did not occur—all estimates should therefore be interpreted as causal under this assumption. We have normalized our results in accordance with this.

Another issue regarding identification should be addressed explicitly. Time-varying, region-specific unobserved variables could potentially be related to both baseline utility store density and children's nutritional outcomes; consequently, the estimated treatment effects will likely contain bias. District fixed effects control for time-invariant heterogeneity among districts and sibling fixed effects control for common household characteristics. Thus, these controls reduce the number of possible confounding variables. On the other hand, there may still exist unobserved variables that vary both over time and across regions, especially those that differentially impact districts receiving larger subsidy shocks. Although Section 5.6 reports that the results of robustness tests support the validity of the identification assumptions made in this study, these tests cannot completely rule out the presence of such variables. Therefore, the causal inference drawn from the estimates depends upon the assumption that, had no reform occurred, child nutrition trajectories would have remained similar (parallel) in districts with high levels of exposure and low levels of exposure — an assumption supported by the event study analysis but not directly tested.

A potential tension in our framework merits explicit discussion before proceeding. Section 2 theorized that food subsidies can serve as an instrument of elite political control, creating vested interests that resist reform. Yet our empirical finding is that reducing subsidies—as occurred under IMF pressure in 2008–2010—harmed child nutrition, particularly among poor households. Does this go against the argument that it is an elite tool of subsidies? We argue it does not. The solution is to identify elite intention from incidental effect. The primary purpose of Pakistan's wheat subsidy system was to ensure political compliance from big farmers, urban consumers and patronage networks—not to improve child health. The political logic of these subsidies is not undermined by the fact that they also provided incidental nutritional benefits to poor households. When subsidy reduction was pushed through under external pressure (IMF conditionality), the burden of adjustment fell more heavily on those least able to adjust: these are poor households with small children. So, we see a kind of tragic irony: a policy instrument for elite control that is subject to dismantling ends up doing disproportionate harm to the very people whose nutritional deficiencies may ultimately — and paradoxically — help reinforce elite power by reducing party capacity. This isn't a contradiction in logic, but a description of how inept political equilibria can hang on. Elites allow some pecuniary gains to the poor as a side effect of their own rent-seeking, but when those rents are endangered (by fiscal crises) they make the poor pay for the adjustment.

Our results contribute to a growing literature on the political economy of agriculture by demonstrating how what appear to be purely fiscal or economic policies can have intergenerational effects through biological pathways. Although basic research has investigated the political motivations and economic inefficiencies of agricultural subsidies (4, 36), we find that these policies may have a transformative effect on the human capital foundation of future political structures. The finding that subsidy cuts negatively impacted child nutrition in districts depending on these supports corroborates previous work emphasizing the special sensitivity of early-life diets to price variation (92, 93). Further, the lingering effects of these negative impacts into adolescence and their association with lower civic engagement adds a unique contribution that connects food policy with important questions of state capacity and democratic development (94, 95).

Second, this article contributes to conversations about institutional resilience in new democracies. The link which we see between policy deficits driven by malnutrition, and a smaller civic capacity follows from theoretical arguments that human capital is critical for political accountability as well as collective action (96, 97)—in short it shapes political behavior and state–society relations. To this end, the finding links to debates regarding state capacity micro-foundations (98–100). Given the challenges of institutional fragility for development in Pakistan (101), our findings are consistent with the idea that policies limiting early-life nutrition might destroy the civic functions through which citizens secure better governance. We stress, however, that this interpretation is theoretically suggestive; we do not pretend to have empirically established all of the links in the feedback loop from nutrition to institutional persistence.

Third, our findings add to the literature on the intergenerational transition of economic status and poverty traps. The long-lasting impacts of early nutritional shocks on educational reaches are in line with studies focusing on the enduring scarring consequences of childhood adversity (102, 103). In particular, the shocks that we study are politically-induced—the result of specific, planned reforms enacted under pre-conditions. The finding relates to the institutional basis of inequality where many scholars argue that the political economy of subsidy design may facilitate or depress transgenerational disadvantage (104, 105). Like our other interpretive claims, this is a proposal rather than an assertion of causality.

Fourth, our findings have relevance for the political economy of reform in developing countries. This mention of coercive efficacy identifies the immediate human capital costs of politically imposed adjustment, a reminder not only that structural adjustment is painful, but also requires tough choices and distributional struggles (106–108). The asymmetric effects—source of the highest cost borne by poor households and young children—tests empirical ex ante specific compensation requirements in functional reform design (109, 110). Implications Given our insights, it seems plausible that very specific vulnerable population groups can be targeted while traversing certain transitions of a policy in order to better fashion operational pathway to propagate such safety nets.

The results undermine standard operating procedures for policy evaluation. Traditional/b> (cost-benefit) approaches to the analysis of subsidies or their elimination have been oriented around short-term fiscal savings and allocative efficiency. Nonetheless, our findings suggest that future research should broaden this approach to include lasting inter-generational effects of human development (111, 112), particularly since recent literature has shown extremely high economic returns for investments in nutrition providing early childhood development support. This role is consistent with a larger change toward welfare assessment in conservation economics (113, 114).

In non-experimental designs that are less amenable to experimental design, a difference-in-differences design that uses pre-existing differences in exposure (here infrastructure features) was a reliable method for causal inference (115). The finding from the event study analysis also provides additional confidence in the parallel trend's assumption. We acknowledge some limitations: despite controlling for endogeneity, the problem of possible unmeasured confounders persists (116). Moreover, our civic engagement indicators are based on indirect proxy measures. This could be particularly useful in future research if we were to compare the great place policies that the sample individuals actually encounter, through surveys or administrative data, with their observable and direct political behavior such as voting, petitioning, or attending community meetings (51).

This analysis links economic development with people's political participation. In our research, we have reached a core finding: long-term pressure from undernutrition weakens citizens' ability to participate in public affairs. This conclusion is consistent with existing academic conclusions from previous studies — those studies have long noted that fluctuations and shocks at the economic level can generate impacts on the political sphere (117, 118). Beyond that, we have also sorted out a set of pathways through which nutrition exerts its effects. These pathways have tangible impacts on every individual, not only affecting personal physical development, but also slowing the development of cognitive abilities (102). This newly sorted set of nutrition impact pathways also provides key supporting content for another category of research — that category of research has been discussing how a person's early growth environment shapes, step by step, an individual's social outlook and civic values (119, 120). Additionally, it is important to align with previous studies to support our outcome that the heterogeneous dietary responses observed in this study align with broader evidence that household socio-economic characteristics mediate the capacity to adjust consumption during price or policy shocks (121, 122).

A limitation of our identification strategy warrants attention. Although the 2008–2010 wheat subsidy reform can be considered plausibly exogenous (importantly as it was driven by IMF conditionality induced by global price shocks), our exposure measure, pre-reform utility store density, may also be correlated with unobserved district-level covariates that influence child nutrition trajectories. First, we have addressed this concern in a number of ways: the inclusion of district fixed effects absorbs arbitrary time-invariant confounding; sibling fixed-effects comparisons within households yield nearly identical estimates (Table 2, column 4); placebo tests defining the treatment year produce null effects; and our event study does not reject the parallel trends assumption (Table 3; Figure 4). An ideal design would instrument store density with historical infrastructure (i.e., colonial road networks /distance to provincial capitals), however such data are not available at the district level for Pakistan. Overall, our estimates should therefore be interpreted causally under the assumption that without the reform child nutrition trends would have remained parallel across highlands low-exposure districts—an assumption consistent with our event study evidence but ultimately untestable.

## 7 Conclusion

This paper assesses whether the 2008–2010 reform of Pakistan's wheat subsidy program led to implications for other areas outside of the program's direct impact on fiscal and price consequences. To examine this question, the analysis employs district-level variation in pre-reform exposure to the public distribution system as a means to assess the impacts of the reform using a difference-in-differences framework. The results indicate three main conclusions.

First, after the reforms were implemented, the children who lived in those districts which had a higher amount of exposure prior to the reforms were estimated to experience an average decrease of about 0.28 standard deviations in their height-for-age z-scores. Height-for-age is a commonly used measure of linear growth and nutritional status, and a reduction of this size would represent a large decline in the children's physical development when compared to the WHO reference distribution.

Second, the decline in child nutritional status was associated with significant alterations in the types of foods consumed within households. In districts with high exposure to the crisis, households had less variety in their diets; they also ate fewer protein rich foods, fewer dairy products, and fewer fruits. Additionally, food spending on wheat flour increased in these districts. These results indicate that households may have been able to maintain their caloric intake through the substitution of a higher priced staple food (wheat flour) as the price of that food increased relative to other foods.

The third finding is that there is a negative relationship between early life nutritional shocks and subsequent standardized math performance; and women living in the most heavily affected areas have significantly lower levels of autonomy, mobility, media access and participation in community organizations. It should be noted that the correlations described in the previous sentence are correlational and may be seen as indicators of civic capacity rather than indicators of direct political accountability. Data used to conduct this study does not allow us to establish a complete causal link from child nutrition to political accountability or policy stability.

In summary, the study's results indicate that food policy is more than just a tool to help individuals meet their daily calorie needs; it can be used as a means of enhancing human capital throughout an individual's life. The effects of the subsidy reforms go beyond short term financial and consumer outcomes and affect physical development as well as possibly future cognitive and civic outcomes. This evidence supports combining reforms to staple foods with protective measures for the nutritional needs of young children so that the burden of adjustment does not fall on the shoulders of people who are least able to bear it. Whether nutritional deficiency will allow the continuation of poor policies due to the inability of people to hold their leaders accountable is a question which future research can answer directly.

### 7.1 Specific contribution of this research to health economics and public policy

In current study, there are three ways in which this research contributes to the fields of health economics and public policy are.

First, it creates an analytical model based on the concept of “nutritional lock-in.” It combines three areas of study that are generally analyzed independently: the politics and economics surrounding food and agricultural policies; the developmental origins of health and disease; and the economics of developing human capital. This model establishes a self-reinforcing process whereby elite-created food policies affect the nutrition children receive at birth and throughout their childhoods, and these childhood nutritional states subsequently affect both human capital development and aspects of civic engagement.

Second, it will provide quasi-experimental evidence based on the wheat subsidy reforms that took place in Pakistan during 2008–2010. By utilizing variations in pre-reform exposure to the Public Distribution System (PDS), the research will demonstrate a statistically significant and economically relevant reduction in child height-for-age Z-scores following the implementation of the reforms. It is anticipated that this finding will be robust to child, maternal, and household controls, sibling fixed-effects, and other measures of exposure.

In addition to being linked with long-term consequences such as standardized math performance and measures of women's empowerment, the dietary influences are also linked to other long-term results. Although the relationships described in the previous sentence provide some indication of long-lasting links between the nutritional effects and the subsequent outcomes, they do not establish cause-and-effect relationships.

The policy implications are consistent in each of the three articles: when designing fiscal or food-policy reforms, there must be specific protections designed to safeguard the nutrition of children. Additionally, the research indicates that adjusting to new policies will have a greater impact on low-income families who have young children, and this impact could continue to affect their ability to develop human capital throughout their lives. Therefore, incorporating nutritional safeguards into the design of reforms is not simply about providing assistance to those in need; it is a prerequisite for developing human capital over the long term.

## Data Availability

The data that support the findings of this study are derived from publicly available sources. The primary survey data are from the Pakistan Demographic and Health Surveys (PDHS): 2006–2007; 2012–2013, and 2017–2018 available at (https://dhsprogram.com/data/available-datasets.cfm). Supplementary survey data are from the Pakistan Multiple Indicator Cluster Surveys (MICS) (https://mics.unicef.org/surveys) and the Household Integrated Economic Survey (HIES) (https://www.pbs.gov.pk/content/household-integrated-economic-survey-hies). Additional contextual data are from the annual reports of the Utility Stores Corporation (https://usc.org.pk/downloadfile). Electoral data from the Election Commission of Pakistan (https://www.ecp.gov.pk/) were consulted for contextual purposes only; they are not incorporated as dependent variables in the main analysis due to the unit of analysis mismatch with the district-level PDHS data and the lack of temporal alignment with the cohort-based identification strategy. All data used in the main analysis are publicly available and can be accessed through the sources listed above.

## Appendix

**Table A1:** Correlates of pre-reform utility store density (1998).

Variable	Coefficient	Robust SE
Adult literacy rate (%)	0.006	(0.005)
Population density (log)	0.038^**^	(0.016)
Urban population share (%)	0.028^*^	(0.015)
Wheat production (tons per capita)	−0.009	(0.019)
Constant	0.152	(0.098)
Observations	120	
R-squared	0.31	

significance at **p* < 0.10, ***p* < 0.05, ***p* < 0.01. Standard errors are robust. All variables measured at the district level from the 1998 Population Census.
